# Spatiotemporal Dynamics and Epistatic Interaction Sites in Dengue Virus Type 1: A Comprehensive Sequence-Based Analysis

**DOI:** 10.1371/journal.pone.0074165

**Published:** 2013-09-09

**Authors:** Pei-Yu Chu, Guan-Ming Ke, Po-Chih Chen, Li-Teh Liu, Yen-Chun Tsai, Jih-Jin Tsai

**Affiliations:** 1 Department of Medical Laboratory Science and Biotechnology, College of Health Sciences, Kaohsiung Medical University, Kaohsiung, Taiwan; 2 Department of Laboratory Medicine, Kaohsiung Medical University Hospital, Kaohsiung, Taiwan; 3 Graduate Institute of Animal Vaccine Technology, National Pingtung University of Science and Technology, Neipu, Pingtung, Taiwan; 4 Tropical Medicine Center, Kaohsiung Medical University Hospital, Kaohsiung, Taiwan; 5 Department of Medical Laboratory Science and Biotechnology, College of Medicine and Life Science, Chung-Hwa University of Medical Technology, Tainan, Taiwan; 6 Division of Infectious Diseases, Department of Internal Medicine, Kaohsiung Medical University Hospital, Kaohsiung, Taiwan; 7 Department of Internal Medicine, School of Medicine, College of Medicine, Kaohsiung Medical University, Kaohsiung, Taiwan; University of Rochester, United States of America

## Abstract

The continuing threat of dengue fever necessitates a comprehensive characterisation of its epidemiological trends. Phylogenetic and recombination events were reconstructed based on 100 worldwide dengue virus (DENV) type 1 genome sequences with an outgroup (prototypes of DENV2-4). The phylodynamic characteristics and site-specific variation were then analysed using data without the outgroup. Five genotypes (GI-GV) and a ladder-like structure with short terminal branch topology were observed in this study. Apparently, the transmission of DENV1 was geographically random before gradual localising with human activity as GI-GIII in South Asia, GIV in the South Pacific, and GV in the Americas. Genotypes IV and V have recently shown higher population densities compared to older genotypes. All codon regions and all tree branches were skewed toward a negative selection, which indicated that their variation was restricted by protein function. Notably, multi-epistatic interaction sites were found in both PrM 221 and NS3 1730. Recombination events accumulated in regions E, NS3-NS4A, and particularly in region NS5. The estimated coevolution pattern also highlights the need for further study of the biological role of protein PrM 221 and NS3 1730. The recent transmission of emergent GV sublineages into Central America and Europe mandates closely monitoring of genotype interaction and succession.

## Introduction

An alarming trend in the epidemiological profile of dengue virus (DENV) in recent decades is the dramatic increases in its morbidity and mortality rates and the correlation of its geographic distribution with climate change and globalisation [1,2]. In 2007, the Intergovernmental Panel on Climate Change reported that the mean global temperature has increased by roughly 0.74°C ± 0.18°C in the past century [3]. As DENV is transmitted by insect vectors, incubation time outside of human hosts is climate-sensitive and is especially responsive to temperature and humidity changes [4]. This acute infectious disease is characterized by a widely varying clinical spectrum. In patients with symptomatic dengue, which comprises only 24% of infected persons [5], 94% have dengue fever (DF) characterized by mild and self-limiting febrile illness whereas the remaining 6% progress to dengue hemorrhagic fever (DHF)/dengue shock syndrome [5,6]. In 2012, an estimated 2.5 billion people, over two fifths of the global population, lived in DF-endemic areas, and an additional 120 million people traveled to affected areas at least once [7,8]. Therefore, almost half of the global population is currently at risk for DF. The World Health Organization has estimated that, of the approximately 50–100 million cases of DF that occur annually worldwide, 500,000 cases are DHF. Of the 2.5% of these that are ultimately fatal, most involve children [7,9]. The estimated annual burden of DF is 750,000 disability-adjusted life years, and the total annual cost of treatment in dengue-endemic areas is an estimated US$2 billion [10,11]. Therefore the prevalence of DF is now a major international public health concern.

The DENVs, a single-positive stranded RNA virus, belongs to the Flavivirus genus of the *Flaviviridae* family. The approximately 11-kb viral genome is composed of one open reading frame (ORF) region flanked by two non-translated regions (*5′* and *3′ NTR*). The ORF encodes three structural proteins [core (C), premature (prM), and envelope (E)] and seven nonstructural proteins (NS1, NS2A, NS2B, NS3, NS4A, NS4B, and NS5) (Table S1). Four distinct serotypes, DENV-1−DENV-4, have been identified in this enveloped virus [12]. Transmission of DENV is characterized by alternating serotype outbreaks with 3- to 5-year periodicity and within-serotype outbreaks with 7- to 9-year periodicity [13,14]. Notably, DENV-1 has been the primary serotype identified in recent infections reported in travellers to South America and Europe [2,15,16]. Of the DENV-1 outbreaks reported in Paraguay since 1988, molecular epidemiological studies of variation in the E-protein and in the E/NS1 gene region have revealed three to five DENV-1 genotypes. The most widely used genotyping system, which was proposed by Goncalvez et al., classifies DENV-1 into five geographically distinct genotypes: GI in Southeast Asia [17], GII in Thailand, GIII in Malaysia, GIV in South Pacific, and GV in South America [18–20]. Therefore, a clear understanding of the spatiotemporal dynamics of viral succession during DENV epidemics is essential for identifying trends in DENV transmission.

Phylodynamic analyses can reveal how the phylogenetic signature of a pathogen is shaped by the interacting effects of its genetic variation, transmission, epidemiological characteristics, and selection, especially in RNA viruses [17,21]. Since RNA-dependent RNA polymerase (RdRp) lacks a proofreading mechanism, it is inherently error-prone. Phylodynamic patterns are likely to be affected by viral evolutionary and epidemiological processes [22]. Viral evolutionary processes are determined by genetic variation (e.g., mutation and recombination in DENV) upon which natural selection acts. The main epidemiological distinctions are determined by the relative time course of infection (acuteness and transiency vs. chronicity and persistence) and turnover of host immunity after epidemics [23]. In other words, because phylodynamic processes have shaped phylogenetic tree of a pathogen, their phylogenetic topology may reveal the transmission nature of pathogen. One example is a virus subjected to continual immune-driven selection, in which the tree has a strong temporal topology with short terminal branches indicating lineages with high extinction rates [21,24]. Unlike the transgobal spread of influenza virus, which was characterised by acute, short-term infections with partial cross-immunity, DENV reportedly has a short spatial movement, which results in acute short-term infections with antibody-dependent enhancement (ADE). The tree topology of influenza virus is characterised by a ladder-like trunk and short terminal branches, whereas the tree topology of DENV is characterised by a unique equidistant phylogeny with short terminal branches [12,25,26]. The equidistant topology is a result of the survival of DENV because all prevalent lineages are transient under a selection that enhances host immunity [21]. These findings indicate not only that the cladogenesis of RNA viral pathogens depends on genetic variation but also viral survival depends on prevailing epidemiological and immunological conditions.

Modern phylodynamic analyses provide an improved understanding of infectious virus characteristics, including their emergence, transmission pathways, and evolving virulence. In addition to phylogenetic and spatiotemporal transmission studies, bioinformatics now enable multiple sequence alignment-based study for sequence variation patterns of pathogens [27–29]. The random genetic mutation of the RNA virus has a deleterious impact on the fitness of an organism; the occurrence of a further compensatory mutation may rescue the virus from this fatal condition [30]. Thus, viruses can survive by benefitting from two detrimental but compensative mutations. A clear understanding of variation interactions among sites may provide indispensable insights into the key structures and functional sites of protein. The use of codon-based models to detect signatures of selection and co-evolution has further revealed indispensable information about the structure and functional sites of molecules, which are essential for designing effective diagnostic tools, vaccines and drugs. To elucidate the adaptation, transmission, and population dynamics of DENV-1 and their implications for genetic variation, this study analyzed data for 103 strains of DENV genome sequences that have circulated globally during 1943–2009, including four Taiwan strains and an outgroup strain in each of prototype strains DENV-2 through DENV-4. Population dynamics were reconstructed according to geographic and temporally stamped data for 100 DENV-1 strains. The key variations and sites in the genome were also analysed in terms of selection, recombination and epistatic interaction.

## Materials and Methods

### Ethics Statement

Two 1994 and two 2008 Taiwanese isolates from patients with DF in Kaohsiung Medical University Hospital (KMUH) were randomly selected for analysis. This study was approved by the ethics committees of KMUH (KMUH-IRB-960195). All samples were de-identified and analyzed anonymously.

### Sample collection, RNA purification, cDNA synthesis and sequencing

The samples were cultured in C6/36 cells and maintained at 28°C in Delbecco modified Eagle medium (DMEM, Gibco-BRL, Gaithersbury, MD) supplemented with 2% fetal bovine serum (Gibco-BRL). The inoculated cells were harvested and identified by using a direct immunofluorescence antibody. Positive cells were then frozen and thawed several times and then centrifuged. The supernatant was then stored at -80°C until use. Viral RNA was extracted from the viral stock with the QIAmp viral RNA mini kit (Qiagen, Germany) according to manufacturer instructions (Qiagen, Chatsworth, CA, USA). The RNA was eluted in 50μl of RNase free water and stored at -80°C until use. Reverse transcription (RT) and polymerase chain reaction (PCR) were performed as previously described [31,32]. Briefly, fragments of PCR products were amplified with the primer sets as shown in Table S2. Cycle sequencing was performed using the purified PCR products with the ABI Prism Ready Reaction Dideoxy Terminator cycle sequencing kit (Model 3730, Version 3.4, Applied Biosystems, Foster City, CA, USA). The sequences for forward and reverse strands were obtained simultaneously and then edited with Sequence Navigator 3.01 software (PE Applied Biosystems, Foster City, CA, USA).

### Model selection and recombination detection

All sequence data available from GenBank were used for sequence characterization. After excluding the strains with nonsense mutation, the alignment was manually corrected, and ambiguously aligned codons were manually removed. After labeling each strain with the year and country of isolation based on GenBank data or on data in the literature, 37 isolations were identified. For each country, the years from the first to the most recent isolation were divided into 5-year periods, and each strain was assigned to the appropriate period based on its isolation year. Next, 1-5 strains were randomly sampled from each 5-year period. The final analysis included 103 strains of the DENV genome (10,217 nucleotides, corresponding to residues 56–10,372), two randomly chosen Taiwan DENV-1 strains for each of years 1994 and 2008, 96 DENV-1 strains, and prototype strains of DENV-2−DENV-4 as outgroups (Table S3). The Clustal X2.0 multiple sequence alignment program [33] provided by the EMBL-EBI (http://www.ebi.ac.uk/Tools/msa/clustalw2/) was used for multiple alignments. To ensure the broadest area coverage, the 96 DENV-1 strains were proportionally and randomly selected from representative pool of years for each country of origin. The most suitable nucleotide substitution model was identified by jModelTestv0.1.1 [34]. The model with the best fit was then used for recombination, phylogenetic and selection analysis. Recombination detection in the 103 DENV sequences dataset was estimated using the Simplot v 3.5.1 [35] and the RDP v 3.44 [36] software packages.

### Phylogenetic analyses

The MEGA5 software was used to construct the neighbour joining (NJ) and maximum likelihood (ML) tree [37]. The nodal reliability of the NJ and ML trees was assessed by bootstrap (BS) with 1000 pseudo-replicates. The programs used for Markov chain Monte Carlo (MCMC) tree analysis were MrBayes v. 3.1.2 [38] and Bayesian Evolutionary Analysis Sampling tree (BEAST) v.1.5.4 [39]. Regular generations were sampled until convergence was reached. The stationarity of the post burn-in distributions and the estimated parameters were then used to calculate effective sample size (ESS) with Tracer v.1.4 program (available at http://beast.bio.ed.ac.uk/Tracer). Convergence of the MCMC sample on the posterior distribution was defined at an ESS value > 200. The maximum clade credibility (MCC) tree was constructed using TreeAnnotator v.1.4.8 and then visualized using FigTree v.1.3.1 (available at http://tree.bio.ed.ac.uk/software/figtree/). The nodal support for these analyses was estimated by posterior probability (PP) values.

### Demographic and spatiotemporal dynamic reconstruction

For real-time analysis of spatiotemporal dynamics and for statistical efficiency, the Bayesian statistical inference framework was implemented in the BEAST package. Since BEAST can implement several combinations of demographic scenarios and clock models, approximate marginal likelihoods were calculated for twelve coalescent demographic models, including parametric models (constant population size, exponential growth and logistic growth) and Bayesian skyline plot (BSP) with strict, uncorrelated lognormal distribution (ucld) and uncorrelated exponential distribution (uced) relaxed molecular clocks [39]. The model with the highest BF for marginal likelihood according to the TRACER program was considered the most suitable composition. A co-estimate of the rate of growth (*r* = *Ne.g*) (i.e., the effective number of transmission events per pathogen generation time), the substitution rate (substitutions/site/year), and mean time to most recent common ancestor (TMRCA) were calculated in the BEAST runs of MCMC. Each estimated parameter was indicated by its value and the 95% highest probability (HPD). An MCC tree with the probable location states of each cluster was constructed using TreeAnnotator. The major routes of geographic diffusion were identified by using the Rate Indicator BF tool in the BEAST package to analyze each rate in two different locations indicated by latitude and longitude. The tree was converted into a keyhole markup language file on the SPREAD application [40] and GeoPhylo website (http://geophylo.appspot.com/) [41].

### Variation, selection and epistatic interactions among DENV-1

A distance matrix based on the genome sequences in the 100 DENV-1 strains and in the three outgroup strains was constructed with MEGA5. The ratio of transitions vs. transversions (Ts/Tv) and frequency distributions of pairwise comparisons were also calculated. The 3392 codon sequences (CDS) of the 100DENV strains were also analyzed to determine site-specific substitution (Hi), evolution rates and selection pressures (ω = *dN*/*dS*). The substitution rates over sites were expressed as an entropy-based measurement and were visualized by plotting sequence variability. The *Hi* values were obtained by calculating the entropy (H) at site *i* (*Hi*) in Data Analysis and Molecular Biology and Evolution (DAMBE) v.5.0.80 [42], and a *Hi* value exceeding 0 indicated the occurrence of substitution. Selection pressure ω was expressed as the ratio of synonymous (silent, *dN*) to nonsynonymous (amino acid-altering, *dS*) mutation. The ω is an important indicator of selective pressure at the codon level, which indicates the functional constraint on maintenance of the encoded protein. A ω greater than 1 indicates a positive (or diversifying) selection, a ω equal to 1 suggests a neutral mutation, and a ω less than 1 suggests a negative (or purifying) selection. A simultaneous positive value for *dN* -*dS* indicates an overabundance of nonsynonymous substitutions. When determining site-specific selection pressures, the methods used to estimate ω values for each coding site included SLAC, FEL, and MEME [43,44]. The genetic algorithm GA-branch was used to determine branch-specific ω values [45], and the Spider monkey program was used to detect epistatic interactions between sites [28]. To infer conditional evolutionary dependencies of sites in this DENV-1 alignment, a Bayesian graphical model (BGM) is deduced based on sites with significant association according to the reconstruction substitution history revealed by ML-based phylogenetic methods. All previous programs for evolutionary detections were run through the Datamonkey website [27]. A significant association between two sites was defined as a PP exceeding a default cutoff of 0.5. Each meaningful protein structure detected was predicted using Iterative Threading ASSEmbly Refinement (I-TASSER) [46] and Homology/analogY Recognition Engine, Version 2.0 (Phyre2) [47] servers. Three-dimensional molecular graphs were constructed and aligned using PyMOL program (DeLano, WL. The PyMOL Molecular Graphics System, 2002).

### Accession numbers of nucleotide sequences

Sequence data reported in this paper will appear in the DDBJ/EMBL/GenBank databases under accession numbers AB608786-AB608789.

## Results

### Model selection and recombination detection

To understand the roles of recombination and selection in the evolution of DENV-1, two gap-stripped sequence datasets among 100 DENV-1 strains with (10,099 nt) and without (10,316 nt) an outgroup were analyzed, respectively. For both datasets, the best-fit substitution model was the general time reversible model with the shape parameter of a gamma distribution and the proportion of invariable sites in the alignment (GTR+G+I) model (G = 1.7530, I = 0.5330 without outgroup and G = 0.9510, I = 0.3740 with outgroup). Genome-wide comparisons of all 103 sequences were performed to screen the recombination events. Eight mosaic relationships were confirmed in RDP program (Dataset S1). The main recombination breakpoints were located in regions E, NS3-NS4A, and particularly NS5. Interestingly, many strains were defined as major or minor recombination donors in each recombinant event, and, in most recombination donors, both the major and minor donors belonged to the same genotype. This probably resulted from either sequence homology in the donors or from recombination in the ancestor before evolution of the viral subgenotype. The recombination relationships among the mosaic strains, the reference sequences, and the major and minor donors were further confirmed using the SimPlot program (Figure S1). Multiple recombination events were found in FJ196847_97_CN_GD01. Except for AB074751_88_ID_A88 and FJ196847_97_CN_GD01, which displayed the same recombination event in nt 7453–8336 (NS 5), with only one recombinant strain in each recombination event. This might be due to the diverse sampling of viral strains from different locations in this study.

### Phylogenetic reconstruction and spatiotemporal phylogenetic analysis

**Figure 1:**
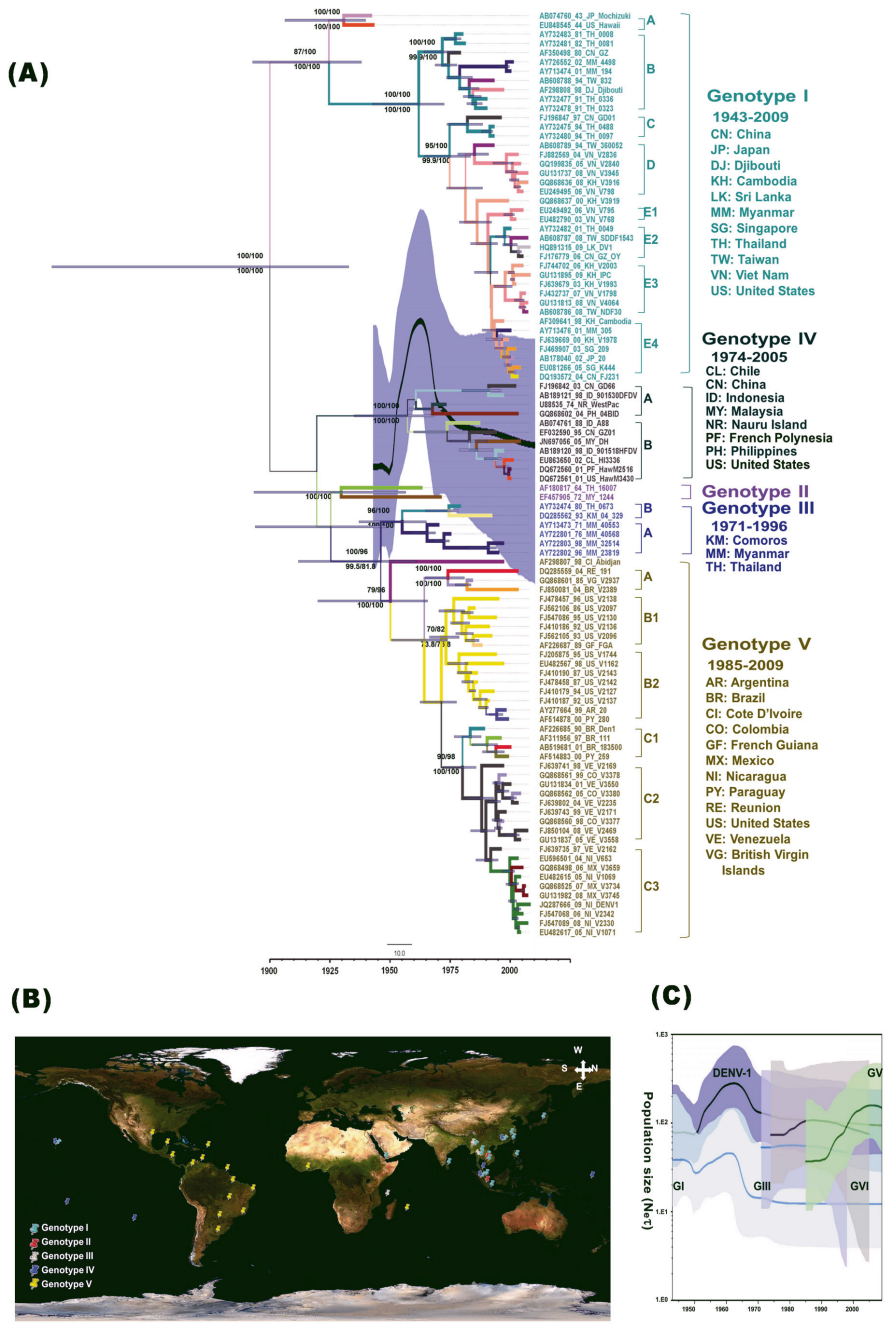
Phylodynamic analysis based on 100 DENV1 genome sequences. (A) Maximum clade credibility (MCC) tree based on 10316 nt gap-stripped MSA, which was constructed using the BMCMC method with BEAST program. The tree shows the proportional relationship between branch length and time; the dashed line below is the scale bar for genetic distance. Each branch thickness indicates the state probability and is colour coded to indicate the most probable locations. Blue bars at nodes indicate 95% highest probability density (HPD). For major lineages, the bootstrap (BS) values and posterior probability (PP) values for the key nodes are indicated as in BS-NJ/BS-ML above and as in PP-MrBayes/PP-BEAST below. Genotypes and subgenotypes are indicated on the right. The thick solid line indicates the median estimates, and the grey area displays the 95% HPD. (B) Overview of geographic dispersal of DENV-1 obtained by SPREAD software (worldmap available at Central Intelligence Agency). (C) Comparison of Bayesian skyline plot between DENV-1 and each genotype. The *x*-axis is the time-scale in years, and the *y*-axis is a logarithmic scale of *N*e*τ* (where Ne is the effective population size and τ is the generation time).

The NJ, ML and MCMC methods were used to construct the relationships among 100 DENV-1 sequences with and without an outgroup. Bayesian posterior probabilities in MrBayes and in BEAST were inferred based on two runs of 1,000,000 and 45,000,000 generations with a burn-in of 10%. All phylogenetic trees constructed using these three methods revealed a similar topology (data not shown). For an efficient statistical analysis and a clear representation of the spatiotemporal dynamics of DENV-1 over time, isolation times and locations were fixed for the 100 worldwide sequences of DENV-1. Table S2 lists the data sets for all sampling locations (*K* = 37). The GTR+G+I with the relaxed uced clock and BSP model composition had the best support in BF analysis. Figure 1A shows the tree with the highest log likelihood (-63072.9 ± 0.43). The tree depicted five genotypes previously designated GI to GV [18]. Further, some genotypes divided into subgenotypes: GI (5, A−E), GIII (2), GIV (2) and GV (3) (Figure 1A). Other than GII, all genotypes and subgenotypes were supported by BS and PP values larger than 70%. In GII, the sylvatic strain EF457905_72_MY_1244 was clustered together with AF180817_64_TH_16007 in the MCMC tree but not in the NJ or ML trees. All 666 dimensions of the rates [*K* × (*K*−1)/2] were well-supported (BF > 20).

Initially, the virus transmission was relatively random; the highly sparse transmission eventually resulted in a wide distribution of ancient strains before the 1980s. After the 1980s, the spatiotemporal signature showed a discernible geographic niche for each genotype. The GI was distributed mainly in Asia at latitude 10.228°−36.205° and longitude 96.953°−138.253° (Figure 1A, 1B and Table S3). The exceptions included three strains each from Hawaii (isolated in 1944), Djibouti (1998), and Sri Lanka (2009). The spatiotemporal relationship of the prevalent GI lineage showed no discernible pattern. Some geographical strains in isolation for longer than 4 years were clustered into the same branch and formed a ladder-like topology, which suggested the emergence and evolution of local viral strains. For example, the Vietnam strains (2005, 2006 and 2008) were clustered in GI-D, and the Cambodia strains were clustered in GI-E3. In contrast, four Taiwan strains had divided into different the sublineages GI-B, GI-D, GI-E2, and GI-E4. The chronological appearance of different lineages at the same location suggested local co-circulation of different lineages and the importation of new viral strains. Genotype II included only two ancient strains as reported previously. Lineage extinction may have already occurred in GII because GenBank shows no more GII sequences for either the full genome or for the gene region [20,48,49]. Genotype III comprised strains isolated earlier in Comoros (1993), Thailand (1980), and Myanmar (1971–1998). The Myanmar strains, which included four strains isolated over a 25-year period, were again clustered into the same sublineage (GIII-A) with a ladder-like signature. Most genotype IV strains were isolates from south Pacific islands and were clustered into two sublineages. Most GV strains in this genotype had been isolated in America (latitude -34.604° 6.128°; longitude -102.553° 36.954°). The GV strains also included one Abidjan strain (1998) and one Reunion strain (2004). The GV-A included a strain isolated in the British Virgin Islands (1985), Reunion (2004), and Brazil (2004). The GV-B and GV-C clusters showed a clear spatiotemporal signature extending from Central to South America. Unlike GV in the Americas, which showed a strong phylogenetic structure (lower part of Figure 1A-B), the GI-IV strains that co-circulated in Asia displayed a nonstructural phylogeny and no clear transmission pattern (Figure 1A-B).

Comparisons of estimated mean (95% HPD) substitution rates showed that the TMRCA values for DENV-1 with and without an outgroup datasets were 1.50 × 10^-3^ (1.04 × 10^-3^-2.01 × 10^-3^), 6.98 × 10^-4^ (5.51 × 10^-4^-8.45 × 10^-4^) and 1188 (688.2–1581.4), 1894.3 (1835.5–1934.9) substitutions/site/year, respectively. For a clear discrimination, the dataset without an outgroup was used in the following spatiotemporal analysis. TMRCA estimations for each genotype were GI: 1906.0 (1866.5 – 1933.9), GII: 1921.4 (1890.6-1950.2), GIII: 1944.4 (1914.5-1963.7), GIV: 1908.5 (1858.1-1948.1) and GV: 1944.4 (1914.5-1963.7) (Figure 1C). The BSP analysis of demographic trends in DENV-1 transmission revealed a dramatic peak in 1962, which suggested that the virus continued to spread and evolve before dividing into GII−GIII. The virus population sharply declined and then stabilised simultaneously with the transmission of GIV in the South Pacific in 1970s. By the 1980s, GV had become prevalent in the Americas (Figure 1A, 1C). Notably, the GIV and GV have the largest population sizes among all genotypes.

### Sequence diversity and selection analysis in the DENV-1 genome

**Figure 2:**
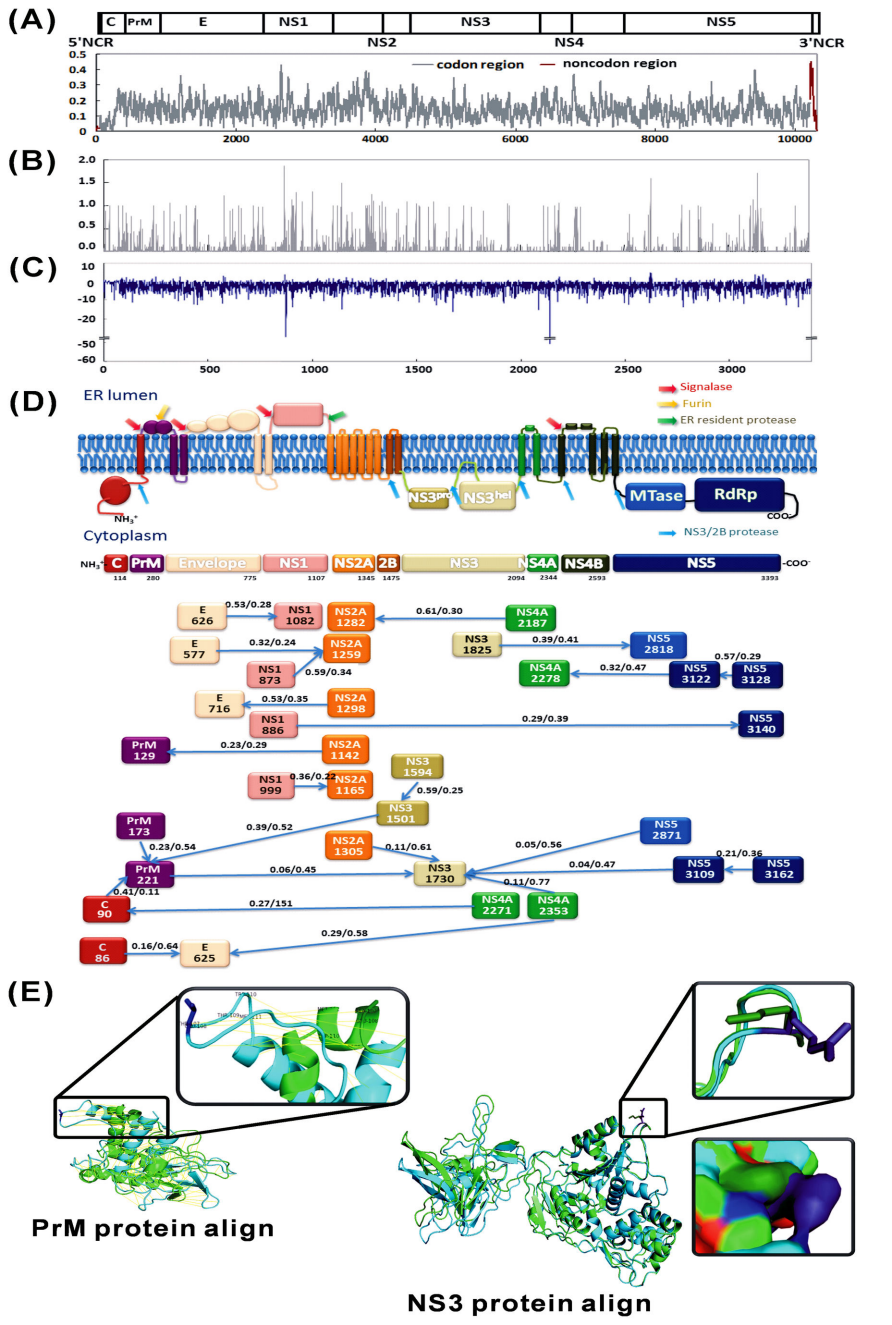
Estimated site-specific variation. The entropy-based variability over sites of (A) 10217 nucleotides and (B) 3392 amino acid residues was analysed using the DAMBE program. X axis: (A) nucleotide position; (B) amino acid position. Y axis: entropy value. Window sizes of 20 and 1 were used in the nucleotide and aa analyses, respectively. (C) Site-specific selection detection. Normalised dN-dS values were plotted for each codon site. (D) Epistatic interaction among DENV-1 codon sequences. In the above graph depicting the codon regions, each square node represents a residue position in the DENV1 codon sequence that participates in at least one interaction. The arrows (edges) representing those interactions are annotated with the fraction of graphs sampled in the chain sample that contains the edge. The analysis reports edges with marginal posterior probabilities (PP) exceeding a default cutoff of 0.5. These data are shown as PP{→}/PP{↔}/PP{←} beside the arrow, which indicates the direction. Both (C) and (D) were performed using the HyPhy software package. (E) Comparison of predicted three-dimensional residue substitution structures in (Left) PrM and (Right) NS3 proteins. The Mochizuki strain, a prototype DENV-1 strain, was used as a template for structural comparison. The wild-type (green) and substitution variant (blue) structures in the figure are aligned and oriented such that the substitution sites are positioned at the top. The substitution sites are highlighted in darker colours. The side chains of substitutions in the PrM^107^ and NS3^255^ are shown. For each substitution, the magnified view (closed box on the right) is oriented for a clear depiction of conformational change due to substitution. An additional magnified surface view of NS3^255^ is shown on the lower right.

Table 1 summarises the sequence variation in each gene region in the 100 DENV-1 genome. Substitution rates varied substantially throughout the genome (Figure 2A). The estimated maximum difference in the 10314 nt sequences was 8.9%. The estimated maximum differences in nt and aa sequences among the codon region of worldwide DENV-1 strains were 4.3% and 3.1%, respectively. The region with the lowest entropy score (i.e., the least variable nt substitutions) was located at 5′ of the capsid protein region while that with the highest entropy score was located at 3′ non-codon region (NCR). Comparison of entropy scores spanning DENV-1 aa sequences showed the lowest variability in the C-terminus of NS3 to the N-termini of NS4A and NS4B. Variability was highest in NS1 and NS5 (Figure 2A, 2B). The 3392 CDSes of 100 DENV-1 were used to analyse their variation, selection and co-evolution (Figure 2B–2D). The Ts/Tv was 9.73 instead of the expected ratio of 0.5 due to random molecular mechanisms. That is, substitution mutations were predominantly transitions, which tend to cause silent mutations in amino acid sequences, by lowering the mutation load. The ω value estimated by SLAC was 0.064. Two dominant negative dN-dS values were found at codons 874 (NS 1) and 2139 (NS4A). Meanwhile, the N-terminus of the capsid protein showed an accumulation of higher positive selection sites (Figure 2C). In addition, all branch-specific ω values determined for the GA-branch were lower than 1 (0.222-0.009). Generally, the skewed Ts/Tv and negative selection indicates that the CDSes tend to conserve their physiochemical properties, which implies recent evolution to a high-fitness condition.

**Table 1:** Sequence diversity and selection detection of each gene region among 100 strains dengue virus type 1.

Genome region	*5′NCR* ^1^	*Capsid*	*PrM* ^2^	*Envelope*	*NS1* ^3^	*NS2A*	*NS2B*	*NS3*	*NS4A*	*NS4B*	*NS5*	*3′NCR*
**Nucleotide**												
Residue sequence	1-38	39-380	381-878	879-2363	2364-3359	3360-4073	4074-4463	4464-6320	6321-6770	6771-7517	7518-10217	10218-10316
Percentage difference^4^	0.6; 7.9	4.1; 7.8	6.3; 11.2	6.3; 9.8	6.1; 10.2	7.9; 13.0	6.7; 11.3	5.6; 9.0	6.6; 12.4	6.1; 10.0	5.6; 8.6	9.1; 20.2
Tt/Tv^5^	3.06	4.92	5.25	9.56	8.52	8.79	9.67	5.72	14.00	10.79	4.81	7.00
**CDS** ^6^												
Omega values^7^	-	0.185	0.074	0.061	0.088	0.101	0.041	0.036	0.059	0.038	0.070	-
EDS^8^	-	1	0	6	2	4	1	3	1	0	10	-
**Amino acid**												
Residue sequence	-	1-114	115-280	281-775	776-1107	1108-1345	1346-1475	1476-2094	2095-2244	2245-2493	2494-3393	-
Percentage difference^4^	-	3.2; 8.0	1.8; 6.0	2.1; 5.3	2.2; 6.0	4.5; 9.7	2.1; 6.9	1.2; 3.1	2.2; 6.7	1.3; 3.3	1.6; 3.2	-

1 NCR: Non-codon region2 PrM: Pre-membrane protein.3 NS: Nonstructural.

4 Percentage difference indicated by mean; maximum pairwise difference.

5 Tt/Tv: Transition/Transversion values calculated using MEGA5 program and then annotated with Kimura 2 parameter corrections with gamma distributions.6 CDS: codon sequences.

7 Omega values (ω = dN/dS, nonsynonymous/synonymous rate ratio) estimated by SLAC implemented in Datamonkey website.

8 EDS: episodic diversifying selection. Data are shown as EDS (p = 0.1); each aa position was obtained by a mixed effects model of evolution implanted in the Datamonkey website.

After removing the five recombination strains from dataset, the alignment of 95 DENV-1 genome sequences were further used to analyze their epistatic interaction. Twenty-four pairs of interactions were identified by co-evolution analysis (posterior significance level 0.5 based on the NJ tree), which revealed two sites with multi-epistatic pairs (Figure 2D). One site at NS3^hel^ 1730 (corresponding to the 255th residues in NS3, NS3^255^) had five epistatic interaction pairs (PrM 221, NS2A 1305, NS4B 2353, NS5^RdRP^ 2871 and 3109). The second site, PrM 221 (PrM^107^), had four epistatic interaction pairs (C 90, PrM 173, NS3^pro^ 1501 and NS3^hel^ 1730). The PrM protein contained only A107T substitutions, and the NS3 contained only K255R substitutions. The Mochizuki strain was used as template, and the structure of the wild-type and point substitutions in PrM^107^ and NS3^255^ were predicted by I-TASSER and Phyre2 servers. Similar results were obtained by both websites (Figure 2E). In PrM^107^, a point substitution between hydrophobic (A) and hydrophilic (T) residues caused dramatic structural changes. In NS3^255^, K and R had different protruding side chains; their substitution caused a conformational change in the molecular surface.

## Discussion

This study used genomic data to reconstruct the global phylodynamics of DENV-1 over the past 70 years. Although several genome-wide phylogenetic studies of DENV-1 have been reported recently, most have been limited to local strains [15,50]. Compared to gene region analysis, genome-wide analysis is hampered by insufficiencies in the GenBank database and computer estimation capacity. Over 1000 genome sequences of DENV-1 have been deposited in GenBank. Excluding prototype strain, almost all strains have been submitted after outbreaks. As a result, representative strains have tended to accumulate in limited isolation locations and years. Therefore, each viral strain was sampled proportionately from the full range of isolation locations and years to ensure that the sample was representative of large variations in isolation location and time. This study of dengue virus revealed a ladder-like phylogeny rather than equidistant terminal branches, mainly because it focused on DENV-1 rather than performing a cross-serotype analysis as in previous studies [51,52]. Although cross-serotype analysis provides an important evolutionary overview, intra-serotype analysis is needed for an accurate and detailed phylodynamic characterisation. Actually, a ladder-like (i.e., unbalanced) phylogeny has been interpreted as the hallmark of a strong directional selection driven mainly by immune escape [17]. T his feature is most common in a DENV bearing immune-enhancement property. A “boom-and-bust” pattern has been identified as the cause of rapid variation and selection escape in RNA viral evolution, i.e., a cycle of adaptive radiation followed by trimming via extinction of branches [53]. Short branches in the tree indicated rapid turnover of viral lineages under selection pressure whereas extended branches and branches with ladder-like backbones indicated successful sublineages in which continuous variation and adaptation resulted in displacement of the prevalent sublineage. Meanwhile, compared with the strong spatial structural topology in GV, the nonstructural clustering in GI to GIV indicated co-circulation and close interaction of those genotypes, which merits close observation.

The BSP results in this study (Figure 1A, 1C) indicate that the sudden peak in the demographic diversity of DENV-1 in the 1950s may be associated with diversification of the virus into strains GII-GIV and their subsequent transmission, possibly as a result of increased international travel during the post-war economic boom [54]. The dramatic decline and stabilisation may also be linked to the use of dichlorodiphenyltrichloroethane (DDT) [54]. *Aedes aegypti* re-emerged during a period of intensive urbanisation. The TMRCA estimation results in this study considerably differed between DENV with and without outgroup (1680.6 vs. 1894.0, respectively). However, date estimation of the ancestral nodes showed that the estimated mean age and substitution rate of DENV (with outgroup) (821.6 and 600.4, respectively) were similar to those reported earlier (1.50 × 10^-3^ and 1.78 ×10^−3^, respectively) in [55]. Date estimation of the ancestral nodes revealed that the mean TMRCA (1894.0) and substitution rate (6.98 × 10^-4^) for DENV-1 (without outgroup) were also similar to those reported previously (1908 and 7.5 × 10^−4^, respectively, in [15]), possibly because the sampling of different viral strains resulted in a different molecular model (relaxed vs strict) with the best fit [55]. Apparently, the sampling and model composition affected the calculation of mean TMRCA but did not substantially affect the calculation of substitution rate. Recent studies indicate that strong negative selection can introduce major errors when estimating TMRCAs [17,56]. Since negative selection conserves functional genetic features over time due to selective pressure against deleterious variants, the long-term effects of elimination of the less-fit considerably predate the TMRCAs. The literature suggests that the DENV-1 epidemic probably emerged in humans in the late nineteenth and early twentieth centuries [57]. Because of short viral genome and short duration of viral evolution, the estimated TMRCAs may not be substantially reduced. The DENV-1 dataset without outgroup data might be more suitable for analyzing mean TMRAC since the sequence variation between heteroserotype and time gap in the isolation year for the prototype strain might lower the accuracy of the estimation results.

Although the DENV mutation rates are similar to those of other RNA viruses [58], this and previous studies demonstrate that the genome sequences tend to be fixed [15]. One hypothesis is that protein sequences in DENV might be restricted by the needs of the viral life cycle as the virus shuttles between mammalian and mosquito cells [59]. Transition and negative selection may have important roles in fixing mutations that affects fitness. Continuous genetic variation is also needed for further selection. Recombination reportedly occurs less frequently in *Flaviviridae* compared to other families with positive RNA genome viruses such as enterovirus and HIV [30]. Similar recombinant events in E region have been reported previously in AY227664_99_AR_20 and FJ196847_97_CN_GD01 but with different recombinant donors [15,50], and the reported recombinant donors vary according to the sampled strain. The recombinant patterns still need further independent verification. The main recombination breakpoints in region NS5 has been identified in this study, a copy-choice mechanism driven by viral template-switching during viral replication is currently the most widely accepted model [60]. Since RdRp proteins are encoded in the NS5 region and are required for viral replication [61]. Recombination and mutation are two major variation mechanisms in a single-fragment RNA virus. Gene function restricted the protein sequence in DENV has been shown here and previous studies, survived lineage escape from host’s immune pressure by undergoing genetic recombination from existing sequences should be a reasonable pathway. Notably, recombination can dramatically change the viral genome, viral recombination can lead to the emergence of new outbreak lineages. The effects of viral recombination require continuous surveillance.

The co-evolution results in this study revealed seven epistatic interacting pairs, of these, four displayed direct or indirect interaction with NS3^hel^ (Figure 2D). Codons 2818 and 2871 were located within β nuclear localisation sequences (NLS) and α/βNLS, respectively (Table S1). In DENV-3, the βNLS region of NS5 is known to interact with NS3^hel^ [62]. These NLS regions are part of a system that imports flaviviral proteins into the host nucleus and have crucial roles in replication [63,64]. The NS3^hel^ 1730 (NS3^255^) revealed the most complex epistatic interaction pairs in this study (Figure 2E). The NS3^255^ is located between the Walker A and Walker B motifs, both of which are present in nucleotide-binding protein families, that participates in a wide spectrum of cellular functions such as nucleic acid processing, recombination and repair, such as recA [65]. Epistatic interaction sites generally occur in NS3 and NS5 regions were understandable since their biological functions are essential in viral replication and repair.

## Conclusions

The data obtained by phylodynamic analysis can be used for accurate reconstruction of epidemic scenarios. A ladder-like topology has been depicted as virus bears the nature of immune escape; structural clustering of taxa in GV but a nonstructural clustering of taxa in GI to GIV. The observed co-evolution pattern also highlights the need for improved understanding of the biological roles of PrM 221 and NS3 1730 proteins. The newer genotypes GIV and GV have recently shown higher population densities compared to older genotypes. Interestingly, two distinct sub-lineages of South American origin have recently spread to central America and Europe. Endemic transmission of one sub-lineage occurred in Florida, USA in 2009-2010 (corresponding to GV-C3 in this study) [66]. Another sub-lineage emanating from Madeira (corresponding to GV-C2 in this study) was identified in travelers to Finland [16]. Although no emergent GIV sublineages have been reported, emergent sublineages in areas where GIV is prevalent should be closely monitored because of its close interaction with GI and GIII.

## Supporting Information

Figure S1 **Recombination map of 103 strains of the dengue virus genome.** (A) Bootscan and (B) similarity plots were constructed with SimPlot program. Each curve in the figure compares the query sequence and reference genomes. The Y axis is the (A) percentage of permutation trees and (B) pairwise identity of each pair of the sequence, and the X axis is the alignment position. The comparison excludes positions containing gaps. The analysis was performed with a sliding window of 200 nt with a 20-nt step. Comparison used 90% consensus sequences with 1000 pseudoreplicates. For each recombinant event, their parental strains and recombinant region are shown between (A) and (B). The recombinant parental strains list is shown in Dataset S1. For reference, the serotype of each prototype of the outgroup and the genotype of each DENV1 strain are given before the strain name (e.g., genotype/serotype_ accession number_year isolated (last two digits) _country abbreviation_strain name), and the genome structure of the dengue virus is denoted at the top.
(TIF)

Dataset S1 Recombination events detected in DENV samples.
(XLS)

Table S1 Function and structure of DENV proteins.
(DOC)

Table S2 Primers designed to amplify full-length genome sequences of the DENV-1 isolates.
(DOC)

Table S3 **List of sampled DENV-1 strains and outgroup.**
(DOC)
