# Supplementary figures and images for: Spatiotemporal Dynamics and Epistatic Interaction Sites in Dengue Virus Type 1: A Comprehensive Sequence-Based Analysis
Source: PLoS One. 2013 Sep 9;8(9):e74165. doi: 10.1371/journal.pone.0074165 (PMC3767619; doi:10.1371/journal.pone.0074165)

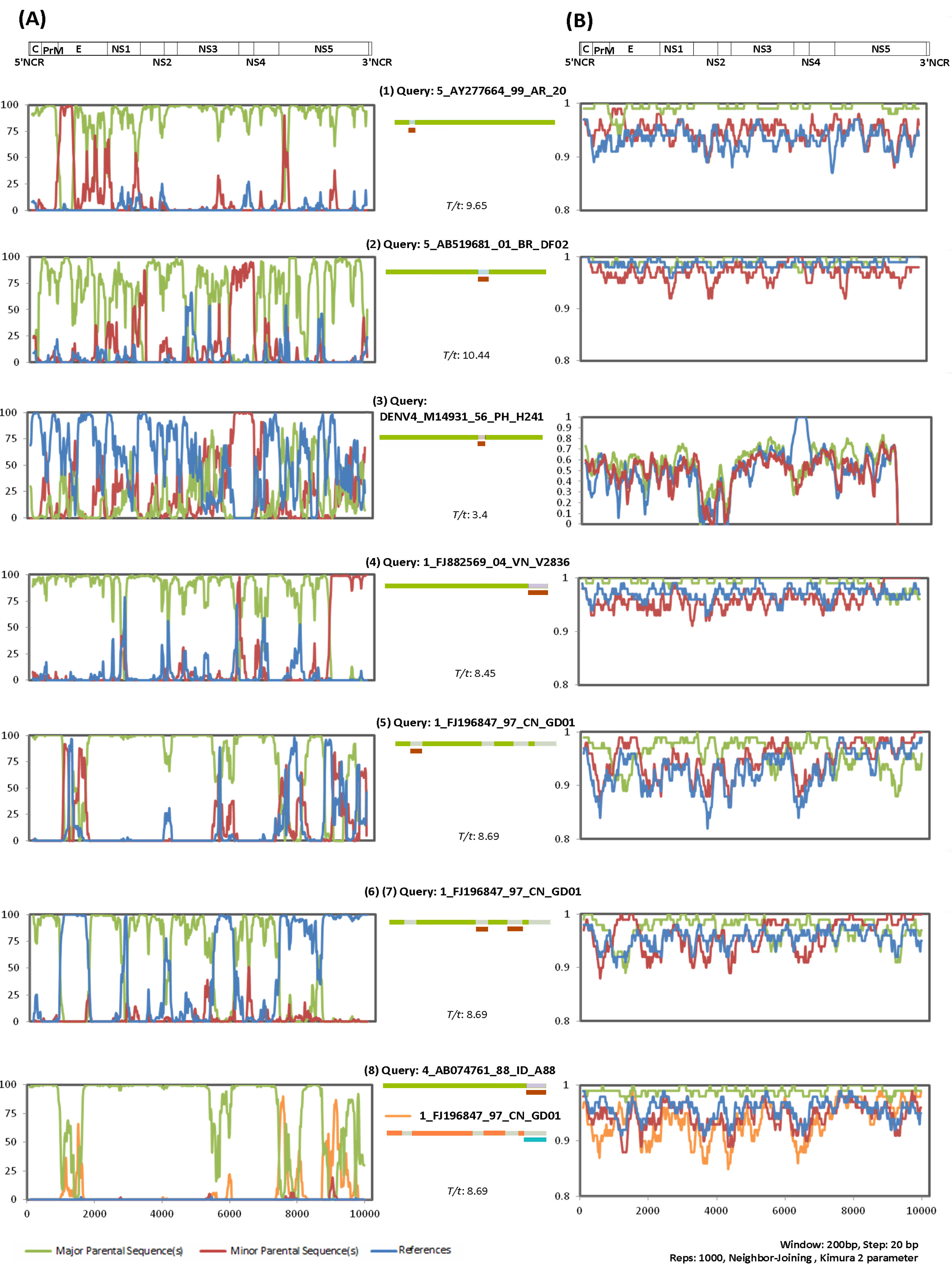

Supplement: Figure S1 — Recombination map of 103 strains of the dengue virus genome. (A) Bootscan and (B) similarity plots were constructed with SimPlot program. Each curve in the figure compares the query sequence and reference genomes. The Y axis is the (A) percentage of permutation trees and (B) pairwise identity of each pair of the sequence, and the X axis is the alignment position. The comparison excludes positions containing gaps. The analysis was performed with a sliding window of 200 nt with a 20-nt step. Comparison used 90% consensus sequences with 1000 pseudoreplicates. For each recombinant event, their parental strains and recombinant region are shown between (A) and (B). The recombinant parental strains list is shown in Dataset S1. For reference, the serotype of each prototype of the outgroup and the genotype of each DENV1 strain are given before the strain name (e.g., genotype/serotype_ accession number_year isolated (last two digits) _country abbreviation_strain name), and the genome structure of the dengue virus is denoted at the top. (TIF) [file pone.0074165.s001.tif]
