# Supplementary material for: Spatiotemporal Dynamics and Epistatic Interaction Sites in Dengue Virus Type 1: A Comprehensive Sequence-Based Analysis
Source: PLoS One. 2013 Sep 9;8(9):e74165. doi: 10.1371/journal.pone.0074165 (PMC3767619; doi:10.1371/journal.pone.0074165)
Supplement: Table S1 — Function and structure of DENV proteins. (DOC) [file pone.0074165.s003.doc]

Table S1. Function and structure of DENV proteins

| Proteins | Functions and special properties | Structure domains |
| --- | --- | --- |
| Capsid | Building block of the protein shell that encloses vRNA, forms the nucleocapsid (NC) core. | - N-terminus (the structural protein with the most variability among the flaviviruses) - Four helices (α1–α4, at aa positions V^26^–K^35^, K^45^–A^58^, A^63^–W^69^, and K^74^–N^96^, respectively) connected by short loops - C-terminus (a signal sequence) |
| PrM | Chaperone for folding and assembly of the E protein | - Pr (~80aa): seven antiparallel β-strands (stabilized by three disulphide bonds), two helices, and two transmembrane helices - furin cleavage site: Arg-X*-(Lys/Arg) -Arg - M peptide (75 aa) |
| Envelope | Attachment and membrane fusion. An E monomer consisting of three β-barrel domains (EDI–III) with folds based largely on β-sheets. The EDIII is the receptor-binding domain. The EDII is a hydrophobic fusion peptide, which is sequence conserved among all flaviviruses. | - EDIII (aa: 1–100): contains BC, DE and FG loops and forms an Ig-like β-barrel structure - EDII (aa: 109–250): a finger-like elongated structure with a hydrophobic sequence in a loop at its tip - EDI (aa: 270–439): located at the N-terminal but with a structurally central domain connecting EDII and EDIII |
| NS1 | An unusual viral glycoprotein not found in infectious virion. It appears in infected cells involved in viral RNA replication and serves as a serum marker of disease severity. | - two N-linked glycosylation sites - 12 hemicystines, and a signalase-like processing site at N-terminus |
| NS2A | Forms part of the RNA replication complex | Contain no conserved motifs characteristic of known enzymes. |
| NS2B | Co-factor for NS3 protease. | Residues 67-80 interact with the NS3^pro^, flanking hydrophobic regions. |

Table S1. Function and structure of DENV proteins (cont’d)

| Proteins | Functions and special properties | Structure domains |
| --- | --- | --- |
| NS3 | Highly conserved multifunctional protein essential for proteolytic activity in N-terminus (serine apoprotease, NS3^pro^) and for RNA replication in C terminus (RNA^hel^). | - NS3^pro^ (~180 aa): consists of six β-strands arranged into two β-barrels. The catalytic center (His^51^, Asp^75^ and Ser^135^) is located between these two β-barrels. - NS3^hel^ (aa: 181–618): subdomain I includes motifs A, B and Ia; subdomain II includes motifs III-VI; subdomain III consists of four α-helices surrounded by three shorter α-helices and two antiparallel β-strands.. |
| NS4A | May induce membrane alterations needed for virus replication. | Contains no conserved motifs characteristic of known enzymes.  A small hydrophobic protein (150 aa, possibly a membrane spanning protein) with a cytoplasmic N-terminal region and rich in α-helices followed by two transmembrane domains separated by a short peptide and a short C-terminal domain. |
| NS4B | May block IFN α/β-induced signal transduction. | A small (248 aa) hydrophobic protein containing no conserved motifs characteristic of known enzymes. |
| NS5 | Methyltransferase (MTase) responsible for capping and methylating the capped + ssRNA Genome on its 5’ terminus and RNA-dependent RNA (RdRp). | - MTase (aa: 1–269): An α/β/β sandwich flanked by N-terminus and C-terminus. - RdRp (aa: 270–900): Composed of six signature-sequence motifs (A-F) and a priming loop. Organized from the N terminus to the C terminus as two nuclear localization sequences (βNLS, aa: 316–368 and α/βNLS, aa: 370–405), finger (comprises codons 273–315, 416–496, and 543–600), palm (aa: 556–639), and thumb (640–798) subdomains. |

*. X: any amino acid
