# Supplementary material for: Spatiotemporal Dynamics and Epistatic Interaction Sites in Dengue Virus Type 1: A Comprehensive Sequence-Based Analysis
Source: PLoS One. 2013 Sep 9;8(9):e74165. doi: 10.1371/journal.pone.0074165 (PMC3767619; doi:10.1371/journal.pone.0074165)
Supplement: Table S2 — Primers designed to amplify full-length genome sequences of the DENV-1 isolates. (DOC) [file pone.0074165.s004.doc]

**Table S2. Primers designed to amplify full-length genome sequences of the DENV-1 isolates**

| Primer | Sequence | Primer position^a^ | Gene region |
| --- | --- | --- | --- |
| 0F^b^ | 5-ACAAGAACAGTTTCGAATCGGA-3 (22mer) | 23–44 | *3*′*NTR/Capsid* |
| 0R^c^ | 5-AGTGACTTTCCTCTTTCCTGCTT-3 (23mer) | 483–505 | *Capsid/prM* |
| 1F | 5-TAAGATTTCTAGCCATACCTC-3 (21mer) | 255–275 | *Capsid/prM* |
| 1R | 5-ATCTCATTAAAGTCTAGCCCTGTT-3 (24mer) | 1521–1498 | *prMen/Env* |
| 2F | 5-GGA ACA ACT GCA ACC ATA ACA CC-3 (23mer) | 1409–1431 | *Env* |
| 2R | 5-TGC CGC TTC CAC ATT TGA GT (20mer) | 2474–2455 | *Env/NS-1* |
| 3F | 5-TCA TGG TTC AGG CGG ACT CG-3 (20mer) | 2406–2425 | *NS-1* |
| 3R | 5-CTA CAG ATG CCA CCA GAC TCA AT-3 (23mer) | 3818–3796 | *NS-1/NS2A* |
| 4F | 5-TCA GAC AAG ATG GGG ATG GGA ACA-3 (24mer) | 5152–5130 | *NS2A/NS2B* |
| 4R | 5-CTC ACG GAC TAT GGC TGG AAG GT-3 (23mer) | 4785–4805 | *NS2B/NS3* |
| 5F | 5-GGA ACG CGG GAG AAG AAG TGC-3 (21mer) | 5792–5769 | *NS3* |
| 5R | 5-CTA TTA CCC TGT CGG CTC GGA AGT-3 (24mer) | 5605–5625 | *NS3* |
| 6F | 5-TGT TCC AAG CAT CAA ATC AGG-3 (21mer) | 6738–6716 | *NS3/NS4A* |
| 6R | 5-GGA ATA AGC AAC ACC ATC AGA AA-3 (23mer) | 6620–6642 | *NS3/NS4A* |
| 7F | 5-TCC ATT GGC CTA CTC TGC GTG AT-3 (23mer) | 8051–8028 | *NS4A/NS4B* |
| 7R | 5-TTC CTT CTT CTA TAG TTG GGT TCG-3 (24mer) | 7953–7971 | *NS4B/NS5* |
| 8F | 5-ACT CCG GGA AAG ATG TAT T-3 (19mer) | 9042–9023 | *NS5* |
| 8R | 5-AAA CCA AGG GCT TCA AAC TC-3 (20mer) | 8793–8816 | *NS5* |
| 9F | 5-CTA TTG GAG CAG TGT TCG TTG ATG-3 (24mer) | 9805–9786 | *NS5* |
| 9R | 5-TCC GGC GCC TTG TGA TAC TC-3 (20mer) | 9318–9340 | *NS5* |
| 10F | 5-CAG CGA AAA ATG GAA CCG TGA TG-3 (23mer) | 9318–9340 | *NS5* |
| 10R | 5-TGT GCC TGG AAT GAT GCT GTA GAG-3 (24mer) | 10692–10669 | *NS5/3*′*NTR* |
| 11F | 5-ATGTGCCACATGTGTTTACAATA-3 (23mer) | 8909–8931 | *NS5/3*′*NTR* |
| 11R | 5-CATTCCATTTTCTGGCGTTCTGT-3 (23mer) | 10689–10711 | *NS5/3*′*NTR* |

^a^ Numbering system used in Ishak *et al*. for DENV-1 prototype Mochizuki strain.

^b^ Sense primer.

^c^ Anti-sense primer.
