# Supplementary material for: Spatiotemporal Dynamics and Epistatic Interaction Sites in Dengue Virus Type 1: A Comprehensive Sequence-Based Analysis
Source: PLoS One. 2013 Sep 9;8(9):e74165. doi: 10.1371/journal.pone.0074165 (PMC3767619; doi:10.1371/journal.pone.0074165)
Supplement: Table S3 — List of sampled DENV-1 strains and outgroup. (DOC) [file pone.0074165.s005.doc]

**Table S3. List of sampled DENV-1 strains and outgroup**

|  | Accession No. | Isolation year | Strain name | Abb.^*^ | Isolation location | Longitude | Latitude | Geno- type |
| --- | --- | --- | --- | --- | --- | --- | --- | --- |
|  | [AB074760](http://www.ncbi.nlm.nih.gov/nuccore/AB074760) | 43 | Mochizuki | JP | Japan | 36.205 | 138.253 | I-A |
|  | [AB178040](http://www.ncbi.nlm.nih.gov/nuccore/AB178040) | 02 | 20 | JP | Japan | 36.205 | 138.253 | I-E4 |
|  | [AB608788](http://www.ncbi.nlm.nih.gov/nuccore/AB608788) | 94 | 832 | TW | Taiwan | 22.918 | 120.432 | I-B |
|  | [AB608789](http://www.ncbi.nlm.nih.gov/nuccore/AB608789) | 94 | 360052 | TW | Taiwan | 22.918 | 120.432 | I-D |
|  | [AB608786](http://www.ncbi.nlm.nih.gov/nuccore/AB608786) | 08 | NDF30 | TW | Taiwan | 22.918 | 120.432 | I-E3 |
|  | [AB608787](http://www.ncbi.nlm.nih.gov/nuccore/AB608787) | 08 | SDDF1543 | TW | Taiwan | 22.918 | 120.432 | I-E2 |
|  | [AF350498](http://www.ncbi.nlm.nih.gov/nuccore/AF350498) | 80 | GZ | CN | China: Guangzhou | 23.129 | 113.264 | I-B |
|  | [EF032590](http://www.ncbi.nlm.nih.gov/nuccore/EF032590) | 95 | GZ01 | CN | China: Guangzhou | 23.129 | 113.264 | IV-B |
|  | [FJ196847](http://www.ncbi.nlm.nih.gov/nuccore/FJ196847) | 97 | GD01 | CN | China: Guangzhou | 23.129 | 113.264 | I-C |
|  | [FJ196842](http://www.ncbi.nlm.nih.gov/nuccore/FJ196842) | 03 | GD66 | CN | China: Guangzhou | 23.129 | 113.264 | IV-A |
|  | [FJ176779](http://www.ncbi.nlm.nih.gov/nuccore/FJ176779) | 06 | GZ_OY | CN | China: Guangdong | 23.132 | 113.267 | I-E2 |
|  | [DQ193572](http://www.ncbi.nlm.nih.gov/nuccore/DQ193572) | 04 | FJ231 | CN | China: Fujian | 26.100 | 119.297 | I-E4 |
|  | [GQ868602](http://www.ncbi.nlm.nih.gov/nuccore/GQ868602) | 04 | 04BID | PH | Philippines | 12.880 | 121.774 | IV-A |
|  | [EU482790](http://www.ncbi.nlm.nih.gov/nuccore/EU482790) | 03 | V768 | VN | Viet Nam: south | 10.228 | 105.601 | I-E1 |
|  | [FJ882569](http://www.ncbi.nlm.nih.gov/nuccore/FJ882569) | 04 | V2836 | VN | Viet Nam: south | 10.228 | 105.601 | I-D |
|  | [GQ199835](http://www.ncbi.nlm.nih.gov/nuccore/GQ199835) | 05 | V2840 | VN | Viet Nam: south | 10.228 | 105.601 | I-D |
|  | [EU249492](http://www.ncbi.nlm.nih.gov/nuccore/EU249492) | 06 | V795 | VN | Viet Nam: south | 10.228 | 105.601 | I-E1 |
|  | [EU249495](http://www.ncbi.nlm.nih.gov/nuccore/EU249495) | 06 | V798 | VN | Viet Nam: south | 10.228 | 105.601 | I-D |
|  | [FJ432737](http://www.ncbi.nlm.nih.gov/nuccore/FJ432737) | 07 | V1798 | VN | Viet Nam: south | 10.228 | 105.601 | I-E3 |
|  | [GU131737](http://www.ncbi.nlm.nih.gov/nuccore/GU131737) | 08 | V3945 | VN | Viet Nam: south | 10.228 | 105.601 | I-D |
|  | [GU131813](http://www.ncbi.nlm.nih.gov/nuccore/GU131813) | 08 | V4064 | VN | Viet Nam: south | 10.228 | 105.601 | I-E3 |
|  | [AF309641](http://www.ncbi.nlm.nih.gov/nuccore/AF309641) | 98 | Cambodia | KH | Cambodia | 12.566 | 104.991 | I-E4 |
|  | [FJ639669](http://www.ncbi.nlm.nih.gov/nuccore/FJ639669) | 00 | V1978 | KH | Cambodia | 12.566 | 104.991 | I-E4 |
|  | [GQ868637](http://www.ncbi.nlm.nih.gov/nuccore/GQ868637) | 00 | V3919 | KH | Cambodia | 12.566 | 104.991 | I-E |
|  | [FJ639679](http://www.ncbi.nlm.nih.gov/nuccore/FJ639679) | 03 | V1993 | KH | Cambodia | 12.566 | 104.991 | I-E3 |
|  | [FJ744702](http://www.ncbi.nlm.nih.gov/nuccore/FJ744702) | 06 | V2003 | KH | Cambodia | 12.566 | 104.991 | I-E3 |
|  | [GQ868636](http://www.ncbi.nlm.nih.gov/nuccore/GQ868636) | 08 | V3916 | KH | Cambodia | 12.566 | 104.991 | I-D |
|  | [GU131895](http://www.ncbi.nlm.nih.gov/nuccore/GU131895) | 09 | IPC | KH | Cambodia | 12.566 | 104.991 | I-E3 |
|  | [FJ469907](http://www.ncbi.nlm.nih.gov/nuccore/FJ469907) | 03 | 209 | SG | Singapore | 1.352 | 103.820 | I-E4 |
|  | [EU081266](http://www.ncbi.nlm.nih.gov/nuccore/EU081266) | 05 | K444 | SG | Singapore | 1.352 | 103.820 | I-E4 |
|  | [AB074761](http://www.ncbi.nlm.nih.gov/nuccore/AB074761) | 88 | A88 | ID | Indonesia: Jakarta | -6.212 | 106.845 | IV-B |
|  | AB189121 | 98 | 901530DFDV | ID | Indonesia: Sumatra | -0.590 | 101.343 | IV-B |

**Table S3. List of sampled DENV-1 strains and outgroup (Cont’d)**

|  | Accession No. | Isolation year | Strain name | Abb.* | Isolation location | Longitude | Latitude | Geno- type |
| --- | --- | --- | --- | --- | --- | --- | --- | --- |
|  | [AB189120](http://www.ncbi.nlm.nih.gov/nuccore/AB189120) | 98 | 901518HFDV | ID | Indonesia: Sumatra | -0.590 | 101.343 | IV-B |
|  | [JN697056](http://www.ncbi.nlm.nih.gov/nuccore/JN697056) | 05 | DH | MY | Malaysia | 4.210 | 101.976 | IV-B |
|  | [EF457905](http://www.ncbi.nlm.nih.gov/nuccore/EF457905) | 72 | 1244 | MY | Malaysia | 4.210 | 101.976 | II |
|  | [AF180817](http://www.ncbi.nlm.nih.gov/nuccore/AF180817) | 64 | 16007 | TH | Thailand | 15.870 | 100.993 | II |
|  | [AY732474](http://www.ncbi.nlm.nih.gov/nuccore/AY732474) | 80 | 0673 | TH | Thailand: Bangkok | 13.752 | 100.494 | III-B |
|  | [AY732483](http://www.ncbi.nlm.nih.gov/nuccore/AY732483) | 81 | 0008 | TH | Thailand: Bangkok | 13.752 | 100.494 | I-B |
|  | [AY732481](http://www.ncbi.nlm.nih.gov/nuccore/AY732481) | 82 | 0081 | TH | Thailand: Bangkok | 13.752 | 100.494 | I-B |
|  | [AY732477](http://www.ncbi.nlm.nih.gov/nuccore/AY732477) | 91 | 0336 | TH | Thailand: Bangkok | 13.752 | 100.494 | I-B |
|  | [AY732478](http://www.ncbi.nlm.nih.gov/nuccore/AY732478) | 91 | 0323 | TH | Thailand: Bangkok | 13.752 | 100.494 | I-B |
|  | [AY732475](http://www.ncbi.nlm.nih.gov/nuccore/AY732475) | 94 | 0488 | TH | Thailand: Bangkok | 13.752 | 100.494 | I-C |
|  | [AY732480](http://www.ncbi.nlm.nih.gov/nuccore/AY732480) | 94 | 0097 | TH | Thailand: Bangkok | 13.752 | 100.494 | I-C |
|  | [AY732482](http://www.ncbi.nlm.nih.gov/nuccore/AY732482) | 01 | 0049 | TH | Thailand: Bangkok | 13.752 | 100.494 | I-E2 |
|  | [AY713473](http://www.ncbi.nlm.nih.gov/nuccore/AY713473) | 71 | 40553 | MM | Myanmar | 21.914 | 95.956 | III-A |
|  | [AY722801](http://www.ncbi.nlm.nih.gov/nuccore/AY722801) | 76 | 40568 | MM | Myanmar | 21.914 | 95.956 | III-A |
|  | [AY722802](http://www.ncbi.nlm.nih.gov/nuccore/AY722802) | 96 | 23819 | MM | Myanmar | 21.914 | 95.956 | III-A |
|  | [AY722803](http://www.ncbi.nlm.nih.gov/nuccore/AY722803) | 98 | 32514 | MM | Myanmar | 21.914 | 95.956 | III-A |
|  | [AY713476](http://www.ncbi.nlm.nih.gov/nuccore/AY713476) | 01 | 305 | MM | Myanmar | 21.914 | 95.956 | I-E4 |
|  | [AY713474](http://www.ncbi.nlm.nih.gov/nuccore/AY713474) | 01 | 194 | MM | Myanmar | 21.914 | 95.956 | I-B |
|  | [AY726552](http://www.ncbi.nlm.nih.gov/nuccore/AY726552) | 02 | 4498 | MM | Myanmar | 21.914 | 95.956 | I-B |
|  | [HQ891315](http://www.ncbi.nlm.nih.gov/nuccore/HQ891315) | 09 | DV1 | LK | Sri Lanka | 7.873 | 80.772 | I-E2 |
|  | [DQ285559](http://www.ncbi.nlm.nih.gov/nuccore/DQ285559) | 04 | 191 | RE | Reunion | -21.100 | 55.600 | V-A |
|  | [DQ285562](http://www.ncbi.nlm.nih.gov/nuccore/DQ285562) | 93 | 04_329 | KM | Comoros | -11.875 | 43.872 | III-B |
|  | [AF298808](http://www.ncbi.nlm.nih.gov/nuccore/AF298808) | 98 | Djibouti | DJ | Djibouti | 11.825 | 42.590 | I-B |
|  | [AF298807](http://www.ncbi.nlm.nih.gov/nuccore/AF298807) | 98 | Abidjan | CI | Cote D'Ivoire: Abidjan | 5.336389 | -4.027 | V |
|  | [AF226685](http://www.ncbi.nlm.nih.gov/nuccore/AF226685) | 90 | Den1 | BR | Brazil: Rio de Janeiro | -22.904 | -43.210 | V-C1 |
|  | [AF311956](http://www.ncbi.nlm.nih.gov/nuccore/AF311956) | 97 | 111 | BR | Brazil: Pernambuco | -8.814 | -36.954 | V-C1 |
|  | [AB519681](http://www.ncbi.nlm.nih.gov/nuccore/AB519681) | 01 | DF02 | BR | Brazil: Brasilia | -14.235 | -51.925 | V-C1 |
|  | [FJ850081](http://www.ncbi.nlm.nih.gov/nuccore/FJ850081) | 04 | V2389 | BR | Brazil: Northern | -3.513 | -49.922 | V-A |
|  | [AF226687](http://www.ncbi.nlm.nih.gov/nuccore/AF226687) | 89 | FGA | GF | French Guiana | 3.934 | -53.126 | V-B1 |
|  | [AF514878](http://www.ncbi.nlm.nih.gov/nuccore/AF514878) | 00 | 280 | PY | Paraguay: Lambaré | -25.346 | -57.610 | V-B2 |

**Table S3. List of sampled DENV-1 strains and outgroup (Cont’d)**

|  | Accession No. | Isolation year | Strain name | Abb.* | Isolation location | Longitude | Latitude | Geno- type |
| --- | --- | --- | --- | --- | --- | --- | --- | --- |
|  | [AF514883](http://www.ncbi.nlm.nih.gov/nuccore/AF514883) | 00 | 259 | PY | Paraguay: Asunci´on | -25.282 | -57.635 | V-C1 |
|  | [AY277664](http://www.ncbi.nlm.nih.gov/nuccore/AY277664) | 99 | 20 | AR | Argentina: Buenos Aires City | -34.604 | -58.382 | V-B2 |
|  | [GQ868601](http://www.ncbi.nlm.nih.gov/nuccore/GQ868601) | 85 | V2937 | VG | British Virgin Islands: Tortola | 18.433 | -64.633 | V-A |
|  | [FJ639735](http://www.ncbi.nlm.nih.gov/nuccore/FJ639735) | 97 | V2162 | VE | Venezuela: Aragua | 10.231 | -67.285 | V-C3 |
|  | [FJ639741](http://www.ncbi.nlm.nih.gov/nuccore/FJ639741) | 98 | V2169 | VE | Venezuela: Aragua | 10.231 | -67.285 | V-C |
|  | [FJ639743](http://www.ncbi.nlm.nih.gov/nuccore/FJ639743) | 99 | V2171 | VE | Venezuela: Aragua | 10.231 | -67.285 | V-C2 |
|  | [GU131834](http://www.ncbi.nlm.nih.gov/nuccore/GU131834) | 01 | V3550 | VE | Venezuela: Aragua | 10.231 | -67.285 | V-C2 |
|  | [FJ639802](http://www.ncbi.nlm.nih.gov/nuccore/FJ639802) | 04 | V2235 | VE | Venezuela: Aragua | 10.231 | -67.285 | V-C2 |
|  | [GU131837](http://www.ncbi.nlm.nih.gov/nuccore/GU131837) | 05 | V3558 | VE | Venezuela: Aragua | 10.231 | -67.285 | V-C2 |
|  | [FJ850104](http://www.ncbi.nlm.nih.gov/nuccore/FJ850104) | 08 | V2469 | VE | Venezuela: Aragua | 10.231 | -67.285 | V-C2 |
|  | [GQ868560](http://www.ncbi.nlm.nih.gov/nuccore/GQ868560) | 98 | V3377 | CO | Colombia: Santander | 6.644 | -73.654 | V-C2 |
|  | [GQ868561](http://www.ncbi.nlm.nih.gov/nuccore/GQ868561) | 99 | V3378 | CO | Colombia: Santander | 6.644 | -73.654 | V-C2 |
|  | [GQ868562](http://www.ncbi.nlm.nih.gov/nuccore/GQ868562) | 05 | V3380 | CO | Colombia: Santander | 6.644 | -73.654 | V-C2 |
|  | [EU596501](http://www.ncbi.nlm.nih.gov/nuccore/EU596501) | 04 | V653 | NI | Nicaragua: Managua | 12.136 | -86.251 | V-C3 |
|  | [EU482615](http://www.ncbi.nlm.nih.gov/nuccore/EU482615) | 05 | V1069 | NI | Nicaragua: Managua | 12.136 | -86.251 | V-C3 |
|  | [EU482617](http://www.ncbi.nlm.nih.gov/nuccore/EU482617) | 05 | V1071 | NI | Nicaragua: Managua | 12.136 | -86.251 | V-C3 |
|  | [FJ547068](http://www.ncbi.nlm.nih.gov/nuccore/FJ547068) | 06 | V2342 | NI | Nicaragua: Managua | 12.136 | -86.251 | V-C3 |
|  | [FJ547089](http://www.ncbi.nlm.nih.gov/nuccore/FJ547089) | 08 | V2330 | NI | Nicaragua: Managua | 12.136 | -86.251 | V-C3 |
|  | [JQ287666](http://www.ncbi.nlm.nih.gov/nuccore/JQ287666) | 09 | DENV1 | NI | Nicaragua: Managua | 12.136 | -86.251 | V-C3 |
|  | [GQ868498](http://www.ncbi.nlm.nih.gov/nuccore/GQ868498) | 06 | V3659 | MX | Mexico: Yucatan | 20.710 | -89.094 | V-C3 |

**Table S3. List of sampled DENV-1 strains and outgroup (Cont’d)**

|  | Accession No. | Isolation year | Strain name | Abb.* | Isolation location | Longitude | Latitude | Geno- type |
| --- | --- | --- | --- | --- | --- | --- | --- | --- |
|  | [GQ868525](http://www.ncbi.nlm.nih.gov/nuccore/GQ868525) | 07 | V3734 | MX | Mexico: Yucatan | 20.710 | -89.094 | V-C3 |
|  | [GU131982](http://www.ncbi.nlm.nih.gov/nuccore/GU131982) | 08 | V3745 | MX | Mexico | 23.635 | -102.553 | V-C3 |
|  | [FJ562106](http://www.ncbi.nlm.nih.gov/nuccore/FJ562106) | 86 | V2097 | US | USA: Puerto Rico | 26.128 | -98.007 | V-B1 |
|  | [FJ410190](http://aeea.nmns.edu.tw/aeea/allyears.html) | 87 | V2143 | US | USA: Puerto Rico | 26.128 | -98.007 | V-B2 |
|  | [FJ478458](http://www.ncbi.nlm.nih.gov/nuccore/FJ478458) | 87 | V2142 | US | USA: Puerto Rico | 26.128 | -98.007 | V-B2 |
|  | [FJ410186](http://www.ncbi.nlm.nih.gov/nuccore/FJ410186) | 92 | V2136 | US | USA: Puerto Rico | 26.128 | -98.007 | V-B1 |
|  | [FJ410187](http://www.ncbi.nlm.nih.gov/nuccore/FJ410187) | 92 | V2137 | US | USA: Puerto Rico | 26.128 | -98.007 | V-B2 |
|  | [FJ562105](http://www.ncbi.nlm.nih.gov/nuccore/FJ562105) | 93 | V2096 | US | USA: Puerto Rico | 26.128 | -98.007 | V-B1 |
|  | [FJ410179](http://www.ncbi.nlm.nih.gov/nuccore/FJ410179) | 94 | V2127 | US | USA: Puerto Rico | 26.128 | -98.007 | V-B2 |
|  | [FJ547086](http://www.ncbi.nlm.nih.gov/nuccore/FJ547086) | 95 | V2130 | US | USA: Puerto Rico | 26.128 | -98.007 | V-B1 |
|  | [FJ205875](http://www.ncbi.nlm.nih.gov/nuccore/FJ205875) | 95 | V1744 | US | USA: Puerto Rico | 26.128 | -98.007 | V-B2 |
|  | [FJ478457](http://www.ncbi.nlm.nih.gov/nuccore/FJ478457) | 96 | V2138 | US | USA: Puerto Rico | 26.128 | -98.007 | V-B1 |
|  | [EU482567](http://www.ncbi.nlm.nih.gov/nuccore/EU482567) | 98 | V1162 | US | USA: Puerto Rico | 26.128 | -98.007 | V-B2 |
|  | [DQ672560](http://www.ncbi.nlm.nih.gov/nuccore/DQ672560) | 01 | HawM2516 | PF | French Polynesia | -17.680 | -149.407 | IV-B |
|  | [EU848545](http://www.ncbi.nlm.nih.gov/nuccore/EU848545) | 44 | Hawaii | US | USA: Hawaii | 19.897 | -155.583 | I-A |
|  | [DQ672561](http://www.ncbi.nlm.nih.gov/nuccore/DQ672561) | 01 | HawM3430 | US | USA: Hawaii | 19.897 | -155.583 | IV-B |
|  | [EU863650](http://www.ncbi.nlm.nih.gov/nuccore/EU863650) | 02 | HI3336 | CL | Chile: Easter Island | -27.121 | -109.366 | IV-B |
|  | [U88535](http://www.ncbi.nlm.nih.gov/nuccore/U88535) | 74 | WestPac | NR | Nauru Island, Western Pacific | -0.550 | 166.917 | IV-A |
|  | [M29095](http://www.ncbi.nlm.nih.gov/nuccore/M29095) | 44 | NGC | PG | New Guinea | -5.500 | 141.000 | DENV2 |
|  | [M93130](http://www.ncbi.nlm.nih.gov/nuccore/M93130) | 56 | H87 | PH | Philippines: Manila | 14.600 | 120.984 | DENV3 |
|  | [M14931](http://www.ncbi.nlm.nih.gov/nuccore/M14931) | 56 | H241 | PH | Philippines: Manila | 14.600 | 120.984 | DENV4 |

* Abb.: Abbreviation for isolation location
